# A Survey on Perspectives Toward Artificial Intelligence Among Italian Interventional Cardiologists

**DOI:** 10.3390/jcm15187050

**Published:** 2026-09-11

**Authors:** Giuseppe Biondi-Zoccai, Giovanni Vincenzo Biondi-Zoccai, Ambra Cerri, Francesco Burzotta, Carlo Trani, Enrico Romagnoli, Arturo Giordano, Nicola Corcione, Salvatore Giordano, Martino Pepe, Carlo Cicerone, Domenico Tavella, Luigi Spadafora, Marco Bernardi, Attilio Lauretti, Francesco Versaci, Simone Calcagno, Fabrizio D’Ascenzo

**Affiliations:** 1 Department of Medical-Surgical Sciences and Biotechnologies, Sapienza University of Rome, 04100 Latina, Italy; gbiondizoccai@gmail.com; 2 IRCCS Maria Cecilia Hospital—GVM Care & Research, 48033 Cotignola, Italy; 3 Dante Alighieri State Classical High School, 00199 Rome, Italy; gvbiondizoccai@gmail.com; 4 Digital Innovation and Development, Fondazione Policlinico Universitario A. Gemelli IRCCS, 00168 Rome, Italy; ambra.cerri@policlinicogemelli.it; 5 Department of Cardiovascular Medicine, Fondazione Policlinico Universitario A. Gemelli IRCCS, 00168 Rome, Italy; francesco.burzotta@gmail.com (F.B.); carlotrani@gmail.com (C.T.); enromagnoli@gmail.com (E.R.); 6 Cardiovascular Interventional Unit, Pineta Grande Hospital, Castel Volturno, 81034 Caserta, Italy; arturgiordano@gmail.com (A.G.); nicolacorcione72@gmail.com (N.C.); 7 Division of Cardiology, Department of Medical and Surgical Sciences, ‘Magna Graecia’ University, 88100 Catanzaro, Italy; sasigiordano@gmail.com; 8 Division of Cardiology, Department of Interdisciplinary Medicine (D.I.M.), Aldo Moro University of Bari, 70124 Bari, Italy; drmartinopepe@gmail.com; 9 Villa Sofia Cardiology Department, AOOR Villa Sofia-Cervello, 90146 Palermo, Italy; c.cicerone@villasofia.it; 10 Division of Cardiology, Department of Medicine, University of Verona, 37126 Verona, Italy; domenico.tavella@aovr.veneto.it; 11 Division of Cardiology, Santa Maria Goretti Hospital, 04100 Latina, Italy; luigispadafora167@gmail.com (L.S.); marco.bernardi23@gmail.com (M.B.); attilio.lauretti@uniroma1.it (A.L.); francescoversaci@yahoo.it (F.V.); 12 Cardiology Unit, Department of Emergency and Admission, San Paolo Hospital, Largo Donatori del Sangue 1, 00053 Civitavecchia, Italy; 13 Division of Cardiology, Cardiovascular and Thoracic Department, Città della Salute e della Scienza, 10126 Turin, Italy; fabrizio.dascenzo@gmail.com

**Keywords:** machine learning, clinical decision support systems, percutaneous coronary intervention, health technology assessment, medical education

## Abstract

**Background:** Artificial intelligence (AI) is increasingly being integrated into cardiovascular medicine, with potential applications across image analysis, procedural planning, risk stratification, decision support, and workflow optimization. However, its adoption in interventional cardiology remains heterogeneous and may be influenced by several factors. We aimed to conduct a nationwide survey to assess attitudes towards AI among Italian interventional cardiologists. **Methods:** We conducted a nationwide, cross-sectional, web-based survey of Italian interventional cardiologists. A structured questionnaire collected information on professional characteristics, familiarity with and current use of AI, perceived clinical applications, expected benefits, trust, implementation barriers, and training needs. Conditional branching was used to obtain additional details from respondents who reported current use of AI-based tools, while all responses were collected voluntarily and analyzed in anonymized, aggregate form. Categorical variables and Likert-scale responses were summarized using descriptive statistics, with exploratory comparisons performed across prespecified professional and institutional subgroups. **Results:** Among 129 respondents, 70.5% reported at least moderate familiarity with AI and 77.5% reported some current use, although only 60.5% reported regular or occasional professional use, and applications were concentrated mainly in research, education, and information synthesis rather than direct procedural support. Nearly half (48.1%) expected AI to become standard in many procedures within 5 years, while 69.0% anticipated either routine use or particular value in complex cases. Attitudes were broadly favorable, with 85.3% agreeing that AI could improve diagnostic and procedural precision, 86.8% expressing strong interest in future use, and 76.7% stating that AI should support rather than replace physician judgment. The leading barriers were medico-legal uncertainty (45.0%), poor integration with existing clinical systems (34.9%), and cultural resistance or operator distrust (29.5%), whereas preservation of physician control was the most frequently cited requirement for adoption (58.9%). Greater AI familiarity was independently associated with current AI use (*p* < 0.001) and good or high trust (*p* < 0.001). Compared with no prior training, one and multiple AI training experiences were independently associated with good or high familiarity (both *p* < 0.05). **Conclusions:** Italian interventional cardiologists showed substantial exposure to AI, strong interest in future adoption, and generally favorable expectations regarding its contribution to diagnostic precision, workflow, and procedural support. Acceptance remained conditional on physician oversight, stronger clinical validation, reliable interoperability, and clear medico-legal governance, and previous AI-focused education appeared independently associated with greater familiarity.

## 1. Introduction

Artificial intelligence (AI), which encompasses several machine learning and deep learning approaches, is increasingly impacting contemporary clinical practice, offering opportunities for automated pattern recognition, risk prediction, and clinical decision support, among many others [1]. Focusing explicitly on cardiovascular care, AI applications have expanded across electrocardiography, multimodality imaging, risk stratification, and workflow optimization, with the broader aim of improving diagnostic precision and supporting personalized management [2]. Despite the obvious challenges inherent to applying AI in an operative clinical subspecialty such as interventional cardiology, this cardiovascular discipline is particularly well suited to novel technologies, as catheterization laboratories generate large volumes of angiographic, physiological, imaging, and clinical data that must often be interpreted rapidly [3]. Within coronary angiography, AI-based systems may facilitate vessel segmentation, automated stenosis detection and quantification, and characterization of lesion complexity or calcification [4]. By standardizing image interpretation and integrating multiple data sources, these tools may also reduce interobserver variability and strengthen procedural planning and decision-making, with obvious benefits for Complex, High-Risk, and Indicated Percutaneous (CHIP) coronary procedures and structural heart disease (SHD) interventions [5].

Clearly, AI may impact coronary physiology and intracoronary imaging by supporting angiography- or computed tomography-derived functional assessment and the automated interpretation of intravascular ultrasound (IVUS) and optical coherence tomography (OCT) [6,7]. In challenging percutaneous coronary intervention (PCI), these tools could integrate anatomical, physiological, imaging, and clinical information to assist procedural planning, device selection, and real-time decision-making [8]. Similar applications are emerging in SHD interventions, where AI may facilitate patient selection, anatomical assessment, multimodality image integration, device sizing, and procedural simulation [9]. Beyond procedural guidance, AI-based models may improve peri-procedural risk stratification, support individualized preventive strategies, and contribute to reductions in radiation exposure, contrast use, and adverse events [10].

Beyond direct procedural support, AI may also favorably impact catheterization-laboratory workflows through automated reporting, registry generation, operational management, research applications, and simulation-based training [11]. However, routine implementation of AI in interventional cardiology practice remains constrained by limited prospective validation, uncertain effects on clinical outcomes, restricted interoperability, financial and organizational barriers, and concerns regarding explainability, privacy, accountability, and preservation of professional autonomy [12]. Given the scarcity of national evidence, this survey aimed to characterize familiarity, current use, trust, perceived benefits, implementation barriers, future priorities, and training needs related to AI among Italian interventional cardiologists.

## 2. Methods

We conducted a nationwide, cross-sectional, web-based survey to assess perspectives toward AI among physicians involved in interventional cardiology in Italy. Eligible participants included practicing interventional cardiologists, catheterization-laboratory directors, fellows in interventional cardiology, and clinical cardiologists with active catheterization-laboratory responsibilities (the details are presented in the Supplementary Materials). Inclusion required current professional activity in an Italian interventional cardiology setting and completion of the Italian-language questionnaire. Responses were excluded when they were incomplete beyond the prespecified threshold, duplicated, submitted by ineligible participants, or otherwise unsuitable for analysis.

Participants were recruited through a nationwide, non-probability sampling strategy targeting physicians involved in interventional cardiology across Italy. The survey invitation was disseminated electronically through professional networks, institutional contacts, and direct email distribution after probing online databases such as PubMed for professional email addresses. A total of 5636 invitations were sent between July 10 and 20 July 2026. Standardized invitation text described the study objectives, expected completion time, voluntary nature of participation, and confidential handling of responses. The questionnaire was administered using an online survey platform and remained accessible from July 10 to 31 July 2026. Reminder communications were distributed at predefined intervals to improve participation while avoiding repeated or coercive contact. No financial or material incentives were offered, and access to the questionnaire required acknowledgment of the study information and provision of electronic informed consent. Duplicate or potentially repeated submissions were screened using standard spreadsheet functions while maintaining respondent confidentiality and complying with applicable data-protection requirements.

The questionnaire was developed from the study objectives, a review of relevant literature and previous physician surveys, and discussion among clinicians and researchers with expertise in interventional cardiology and AI (Table S2). Its content was organized into domains addressing professional and institutional characteristics, familiarity with AI, current use, perceived impact, future expectations, implementation barriers, trust, and educational needs. The instrument included single-choice questions, multiple-response items, five-point Likert-type scales, and selected open-text fields. Questions were designed to be completed within approximately five minutes, with mandatory responses specified for the principal study variables.

Professional variables included age category, primary role, and years of experience in interventional cardiology, while institutional characteristics comprised geographical area, hospital type, catheterization-laboratory profile, and annual procedural volume. Respondents rated their familiarity with AI and reported the availability, frequency, and context of use of AI-based tools. Current applications were assessed across coronary angiography, functional lesion evaluation, intracoronary imaging, procedural planning, risk prediction, workflow optimization, documentation, research, and training. Among users, perceived impact was evaluated in relation to diagnostic quality, procedural planning, safety, efficiency, decision support, operator confidence, and integration into routine practice. Additional items explored expectations for future applications, trust in AI-supported recommendations, perceived benefits and barriers, willingness to adopt these technologies, and educational needs.

Survey data were stored securely and analyzed in anonymized form using the Sapienza University of Rome Google Forms platform, with contact information maintained separately from questionnaire responses where applicable. The primary outcome was defined a priori as current use of AI-based tools, while secondary outcomes included familiarity, trust, perceived benefits, implementation barriers, future expectations, and training needs. Categorical variables are summarized as counts and percentages, whereas ordinal or continuous variables are described using measures of central tendency and dispersion appropriate to their distribution. Exploratory comparisons were performed across prespecified professional and institutional subgroups, including age, experience, geographical area, institution type, catheterization-laboratory profile, procedural volume, and current artificial intelligence use. Categorical variables were compared using Pearson’s chi-square test, Fisher’s exact test, or the Fisher–Freeman–Halton exact test with Monte Carlo estimation, as appropriate. Ordinal variables were compared using the Mann–Whitney U test for two-group comparisons and the Kruskal–Wallis test for comparisons involving more than two groups. Exploratory independent associations were assessed using multivariable binary logistic regression. There were no missing data, as all questions mandated a reply, and thus a complete case analysis was performed. Statistical significance was set at the 2-tailed 0.05 level, without multiplicity adjustment. Computations were performed with Python 3.14.6 (Python Software Foundation, Beaverton, OR, USA). The study was conducted in accordance with applicable ethical and data-protection requirements using the secure IT platform offered by the Sapienza University of Rome, Rome, Italy.

This manuscript was drafted and illustrated with the assistance of artificial intelligence tools, such as ChatGPT 5 (OpenAI, San Francisco, CA, USA), in keeping with established best practices. The final con-tent, including all conclusions and opinions, has been thoroughly revised, edited, and approved by the authors.

Inherently, limitations in the selection process include the non-probability sampling strategy and potential non-response bias. Concerning data processing, data were based on self-reported entries without formal psychometric pre-validation of the survey instrument, and statistical analyses were performed on a strictly exploratory, unadjusted basis.

## 3. Results

A total of 129 physicians involved in interventional cardiology completed the survey (Table 1). Staff interventional cardiologists constituted the largest professional group (38.8%), followed by cath-lab directors or department heads (31.8%), whereas the remaining respondents included clinical cardiologists with cath-lab activity, trainees, residents, and other professional roles. The cohort was highly experienced, with 59.7% reporting more than 10 years of interventional cardiology practice and 38.0% reporting more than 20 years. As expected, professional role was strongly associated with both age and experience, with cath-lab directors representing the oldest and most experienced subgroup.

Self-reported familiarity with AI was predominantly moderate, with 91 respondents (70.5%) reporting at least moderate familiarity and 40 (31.0%) describing their familiarity as good or high. Overall, 100 participants (77.5%) reported some use of AI, including use confined to research or experimental settings. Regular or occasional use in professional activity was reported by 78 respondents (60.5%). The most frequent current applications were scientific research and data analysis, educational support, training and simulation, and intracoronary imaging, whereas direct real-time procedural use was less common. In role-based comparisons, cath-lab directors and staff interventional cardiologists more frequently reported use in intracoronary imaging and functional lesion assessment than respondents in other professional roles.

Among respondents who had used AI, the greatest perceived benefits concerned evidence synthesis and critical review, followed by workflow efficiency, integration into routine practice, and diagnostic quality (Table 2). By contrast, perceived improvements in procedural safety and reductions in procedural time were less common. Overall, 62 respondents (48.1%) expected AI to become standard in many procedures within five years, while 89 (69.0%) anticipated either standard use or particular usefulness in complex cases. The most frequently identified future application was integration of angiographic, intracoronary imaging, and clinical data, followed by clinical research and registry generation, automated reporting, and training applications.

Respondents expressed broadly favorable attitudes toward AI, with 85.3% agreeing that it could improve diagnostic and procedural precision, 74.4% that it could support the training of younger interventional cardiologists, and 71.3% that it could reduce procedural time, radiation exposure, or contrast use. At the same time, 76.7% emphasized that AI should support rather than replace physician judgment, while 62.0% recognized the risk of excessive reliance on algorithmic recommendations. Overall trust was moderate to high, with 49.6% reporting good or high trust and a further 35.7% reporting moderate trust. Interest in future use was particularly strong, as 86.8% of respondents were quite or very interested in using artificial intelligence tools in the coming years. Trust and willingness to adopt supervised artificial intelligence did not differ significantly across professional-role groups.

Medico-legal uncertainty regarding responsibility for AI-related errors was the most frequently reported barrier to implementation (45.0%), followed by poor integration with angiography, PACS, and hospital information systems (34.9%) (Table 3). Other commonly perceived obstacles included cultural resistance or operator distrust (29.5%), insufficient training (27.9%), costs (26.4%), limited clinical evidence (26.4%), and concerns regarding privacy or real-world validation (24.0% each). The principal conditions considered necessary for stable adoption were preservation of physician control over AI recommendations (58.9%), a clear medico-legal framework (44.2%), simple integration with existing systems (40.3%), and validation in large, representative populations (38.0%). Formal educational exposure remained limited, although most respondents supported structured AI training and favored practical, clinically oriented formats such as case-based workshops, fellowships in expert centers, and hands-on congress sessions.

Exploratory subgroup analyses showed age-related differences in selected perceptions and application priorities, while women, who represented 16 respondents, more frequently identified real-time intraprocedural guidance as a priority and loss of physician autonomy as a potential barrier. In multivariable analysis (Table 4), greater self-reported familiarity with AI was independently associated with both current AI use (adjusted odds ratio [OR] 9.91 per category, 95% confidence interval [CI] 3.74–26.26; *p* < 0.001) and good or high trust in AI (adjusted OR 3.26, 95% CI 1.85–5.74; *p* < 0.001). Previous participation in AI-focused education was independently associated with female sex (adjusted OR 4.90, 95% CI 1.22–19.78; *p* = 0.025), younger age (adjusted OR 0.47 per increasing age category, 95% CI 0.22–0.99; *p* = 0.046), and greater AI familiarity (adjusted OR 2.68 per category, 95% CI 1.44–4.99; *p* = 0.002). No independent predictor was identified for comfort with physician-supervised AI recommendations or for expecting AI to assume a clinically relevant role within five years, and all subgroup findings were considered exploratory.

Even after adjustment for age, professional seniority, role, and university-center status, both a single training experience and multiple training experiences remained independently associated with higher AI familiarity. Compared with respondents who had received no training, those trained once had nearly fourfold higher odds of reporting good or high familiarity (adjusted OR 3.98, 95% CI 1.36–11.70; *p* = 0.012), whereas those trained multiple times had approximately elevenfold higher odds (adjusted OR 10.97, 95% CI 2.95–40.82; *p* < 0.001; global *p* for training = 0.0006). Good or high familiarity increased from 19.1% among untrained respondents to 43.5% after one training experience and 76.5% after multiple experiences. The preferred educational formats were practical workshops based on clinical cases, selected by 50 respondents (38.8%); training or fellowships in expert centers, selected by 45 (34.9%); and congress-based hands-on sessions, selected by 43 (33.3%).

## 4. Discussion

In this contemporary survey of Italian interventional cardiologists, most respondents reported at least moderate familiarity with AI, and the majority indicated some current use. Adoption, however, was concentrated in research, data analysis, education, evidence synthesis, and documentation, whereas direct intraprocedural applications and real-time decision support remained uncommon. As also previously reported in another survey on the topic, the greatest perceived benefits similarly involved cognitive and workflow-related tasks, suggesting that AI is entering interventional cardiology through lower-friction informational applications before expanding into more complex procedural settings [12].

Most respondents expected AI to assume a meaningful clinical role within five years, either as a standard component of many procedures or as a particularly useful tool in complex cases. The leading anticipated application was the integration of angiographic, intracoronary imaging, physiological, and clinical data, reflecting demand for more comprehensive and standardized procedural assessment. Other expected applications included automated stenosis quantification, plaque characterization, functional lesion evaluation, complex PCI planning, structural heart procedure simulation, automated reporting, registry generation, research, training, risk prediction, and optimization of radiation and contrast use [13]. These expectations indicate confidence in the AI potential, although prospective evidence remains necessary to establish improvements in outcomes, safety, and workflow efficiency [3]. Importantly, the principal challenge may no longer be clinician acceptance but the ability of healthcare organizations to incorporate validated tools into routine clinical pathways [14]. Indeed, a pivotal finding of our survey is the marked discrepancy between general professional use of AI (e.g., literature synthesis, research data management, and automated reporting) and its direct, real-time integration into interventional procedures. While clinicians readily adopt AI for offline cognitive and administrative tasks, real-time intraprocedural applications, such as live vessel segmentation, automated lesion quantification, or functional decision support, remain far less common. This suggests that the current adoption curve is stepped: lower-friction, non-invasive informational tools are being embraced first, whereas direct procedural integration faces higher barriers related to real-time latency, operator trust, workflow disruption, and legal accountability.

Respondents expressed generally favorable trust in AI and strong willingness to use AI-supported tools under physician supervision. Most preferred an augmentative model in which clinicians retain the ability to confirm, modify, or disregard algorithmic recommendations. This preference emphasizes accountability, contextual interpretation, and professional autonomy amid concerns about excessive reliance, errors, hallucinations, and opaque outputs. Responsible implementation should therefore prioritize human-in-the-loop workflows, transparent communication of uncertainty, and governance models defining how recommendations are validated, documented, monitored, and overridden [15].

Medico-legal uncertainty regarding AI-related errors was the leading barrier, despite recent European regulatory developments such as the EU Artificial Intelligence Act [16]. Limited interoperability with angiography platforms, PACS, electronic health records, and other hospital systems was another major concern [17]. These findings shift attention from individual algorithm performance to the readiness of the organizational ecosystem in which AI must operate, as its clinical value may depend as much on institutional digital maturity, including data quality, information-system architecture, cybersecurity, workflow standardization, and governance, as on technical accuracy [18]. Stable adoption will also require validation in representative real-world populations and prospective evidence of clinical benefit, safety, and cost-effectiveness [19]. Accordingly, investment must extend beyond purchasing AI tools to interoperable infrastructure, reliable data pipelines, institutional oversight, and redesigned workflows [20].

Although formal AI education remained limited, interest in further training was high, particularly for practical workshops, case-based sessions, congress-based hands-on activities, and fellowships in expert centers. Good or high familiarity increased from 19.1% among untrained respondents to 43.5% after one training experience and 76.5% after multiple experiences, supporting repeated, clinically oriented exposure rather than isolated theoretical instruction. Training should encompass not only software operation but also critical appraisal, bias, model limitations, data governance, and safe interpretation of AI outputs, whereas organizational competencies, including pathway redesign, procurement, performance monitoring, incident management, and multidisciplinary collaboration, should also be incorporated [21,22].

Exploratory analyses suggested that cath-lab directors and staff interventional cardiologists more frequently used AI for intracoronary imaging and functional lesion assessment, whereas age- and sex-related differences were limited to selected priorities and concerns. Women more often identified real-time procedural guidance as a priority and loss of autonomy as a barrier, although the small female subgroup precludes robust inference. Greater familiarity was independently associated with current use and higher trust, while previous AI education was associated with female sex, younger age, and greater familiarity. After adjustment, both single and repeated training experiences remained independently associated with good or high familiarity, with an apparent exposure-response relationship. These associations suggest that education may be a modifiable determinant of adoption, although causality cannot be inferred and self-selection remains possible [23]. Furthermore, all multivariable regression and subgroup findings have been strictly framed as exploratory and hypothesis-generating, avoiding overinterpretation of statistical associations within survey-derived data.

The absence of major differences in supervised-AI acceptance across professional groups suggests that implementation barriers may lie more in institutional readiness than in individual attitudes, although limited statistical power cannot be excluded [24]. Scientific societies, training programs, cath-lab leaders, developers, regulators, hospital executives, information-technology teams, and governance bodies should therefore collaborate to develop validated, interoperable, physician-supervised systems. Accordingly, interventional cardiologists advocate for organizational investment rather than isolated acquisition of stand-alone technologies [3,12,25].

Another important aspect to underline is that the substantial participation of cath-lab directors and department heads, representing nearly one-third of our study cohort, provides a particularly relevant organizational perspective. Because unit leaders drive strategic investments, system procurement, and workflow protocols, their high level of interest and familiarity with AI is crucial. Leadership engagement serves as a fundamental catalyst not only for institutional integration and addressing medico-legal frameworks but also for fostering structured training and promoting AI literacy across the entire cath-lab medical and technical staff.

This study is strengthened by its timeliness, national focus, and comprehensive evaluation of AI familiarity, current practice, implementation barriers, and training needs among interventional cardiologists; however, several methodological limitations must be acknowledged. Most importantly, the final sample size of 129 respondents remains limited. While offering valuable preliminary insights, a cohort of this size cannot be assumed to fully reflect or be representative of the entire national interventional cardiology community, and caution is required when generalizing these findings to the broader population of operators. Regarding the selection process, the non-probability sampling strategy and voluntary web-based administration introduce inherent risks of selection and non-response biases. In terms of data measurement and processing, all variables relied on self-reported entries without formal pilot testing or psychometric pre-validation of the survey instrument. Furthermore, given the modest sample size within individual regional strata, granular comparisons across geographical macro-regions lacked adequate statistical power. Finally, statistical sub-analyses and multivariable models were descriptive and exploratory in nature, performed without adjustment for multiplicity, and cannot establish causal relationships, such as that between prior educational exposure and higher self-reported familiarity. Future prospective, longitudinal implementation studies are required to evaluate institutional digital maturity, system interoperability, governance, cost-effectiveness, and real-world clinical outcomes.

**Figure 1:**
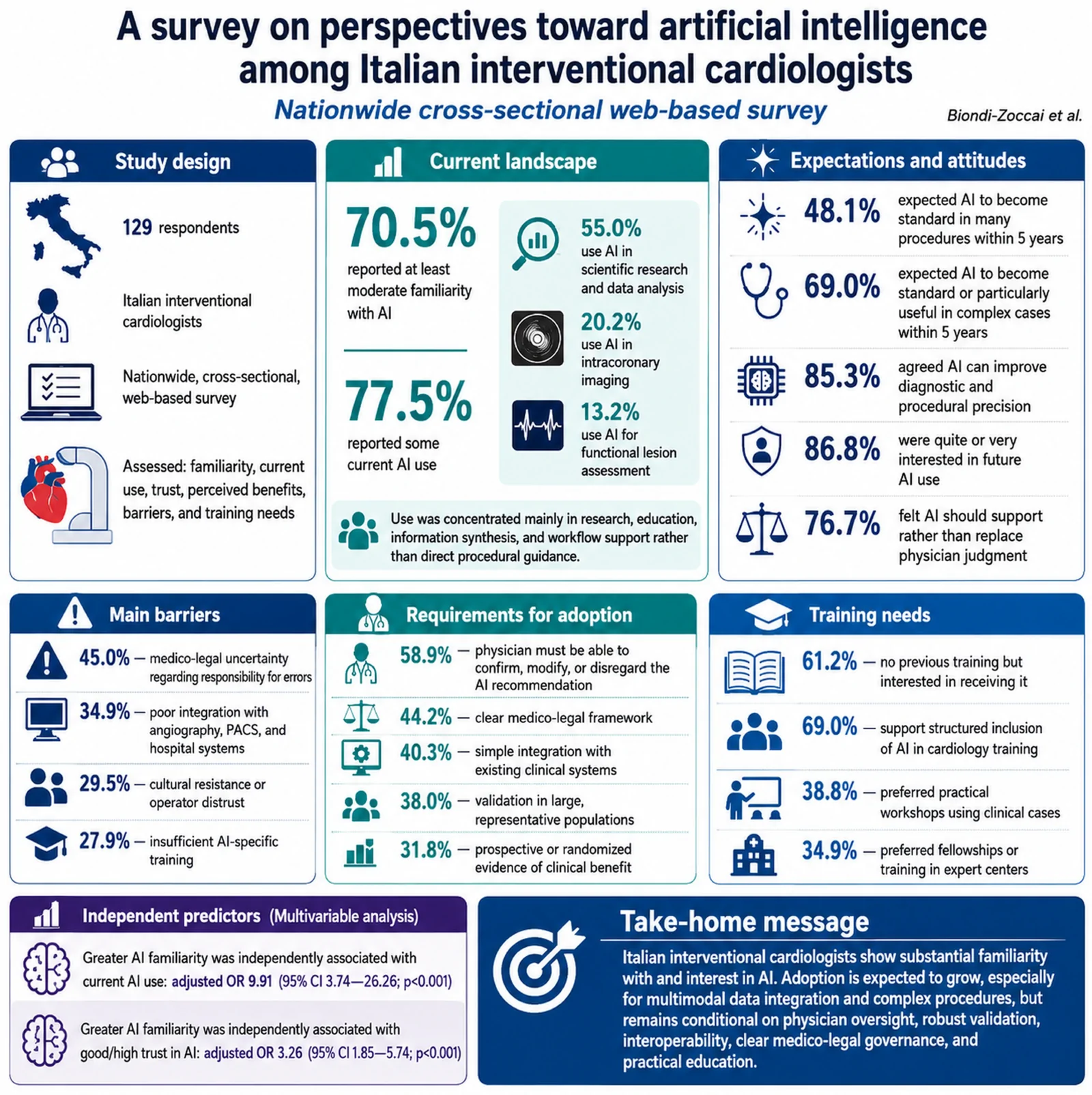
Attitudes toward artificial intelligence among Italian interventional cardiologists.

In conclusion, Italian interventional cardiologists demonstrated substantial familiarity with and interest in AI, although current use remains concentrated in research, education, information synthesis, and workflow support rather than direct procedural guidance (Figure 1). The most distinctive finding is the clear identification of the conditions required for transition into routine care: integration with existing systems, robust clinical validation, transparent governance, defined medico-legal responsibility, and preservation of physician control. The observed relationship between training and familiarity further supports education as a potentially important implementation strategy. Ultimately, successful adoption will depend not only on better algorithms but also on digitally mature organizations capable of integrating them safely, transparently, and sustainably into clinical workflows.

## Figures and Tables

**Table 1:** Participant characteristics, familiarity, and current use of artificial intelligence by professional role.

Variable	Cath-Lab Director/Department Head	Staff Interventional Cardiologist	Other Roles	Overall	*p* Value
Participants	41	50	38	129	
Female	3 (7.3%)	4 (8.0%)	9 (23.7%)	16 (12.4%)	0.065
Age group					<0.001
<35 years	0 (0.0%)	8 (16.0%)	8 (21.1%)	16 (12.4%)	
35–44 years	2 (4.9%)	24 (48.0%)	19 (50.0%)	45 (34.9%)	
45–54 years	16 (39.0%)	10 (20.0%)	5 (13.2%)	31 (24.0%)	
55–64 years	18 (43.9%)	8 (16.0%)	2 (5.3%)	28 (21.7%)	
≥65 years	5 (12.2%)	0 (0.0%)	4 (10.5%)	9 (7.0%)	
Interventional cardiology experience					<0.001
<5 years	0 (0.0%)	8 (16.0%)	18 (47.4%)	26 (20.2%)	
5–10 years	1 (2.4%)	15 (30.0%)	10 (26.3%)	26 (20.2%)	
11–20 years	7 (17.1%)	17 (34.0%)	4 (10.5%)	28 (21.7%)	
>20 years	33 (80.5%)	10 (20.0%)	6 (15.8%)	49 (38.0%)	
Good or high familiarity with AI	14 (34.1%)	15 (30.0%)	11 (28.9%)	40 (31.0%)	0.838 *
Regular or occasional AI use	27 (65.9%)	32 (64.0%)	19 (50.0%)	78 (60.5%)	0.343 *
AI use in research or experimentation only	8 (19.5%)	7 (14.0%)	7 (18.4%)	22 (17.1%)	
Institution lacks AI tools	6 (14.6%)	9 (18.0%)	7 (18.4%)	22 (17.1%)	
Current use in intracoronary imaging	12 (29.3%)	13 (26.0%)	1 (2.6%)	26 (20.2%)	0.005
Current use for functional lesion assessment	9 (22.0%)	7 (14.0%)	1 (2.6%)	17 (13.2%)	0.039
Current use in scientific research and data analysis	28 (68.3%)	25 (50.0%)	18 (47.4%)	71 (55.0%)	0.115

* *p* values marked with an asterisk were calculated using Fisher’s exact test; all other categorical comparisons used the χ^2^ test.

**Table 2:** Expectations, attitudes, trust, and willingness to adopt artificial intelligence by professional role.

Variable	Cath-Lab Director/Department Head	Staff Interventional Cardiologist	Other Roles	Overall	*p* Value
Participants	41	50	38	129	
AI expected to become standard or particularly useful in complex cases within 5 years	30 (73.2%)	35 (70.0%)	24 (63.2%)	89 (69.0%)	0.642
AI can increase diagnostic and procedural precision *	33 (80.5%)	43 (86.0%)	34 (89.5%)	110 (85.3%)	0.687
AI should support rather than replace physician judgment *	26 (63.4%)	41 (82.0%)	32 (84.2%)	99 (76.7%)	0.556
AI can reduce procedural time, radiation, or contrast use *	28 (68.3%)	34 (68.0%)	30 (78.9%)	92 (71.3%)	0.435
AI can improve procedural standardization *	28 (68.3%)	33 (66.0%)	28 (73.7%)	89 (69.0%)	0.393
AI may encourage excessive reliance on system recommendations *	28 (68.3%)	28 (56.0%)	24 (63.2%)	80 (62.0%)	0.329
AI will significantly modify the interventional cardiologist’s role *	17 (41.5%)	15 (30.0%)	20 (52.6%)	52 (40.3%)	0.030
Comfortable using physician-supervised AI recommendations *	28 (68.3%)	32 (64.0%)	18 (47.4%)	78 (60.5%)	0.247
Good or high overall trust in AI	23 (56.1%)	22 (44.0%)	19 (50.0%)	64 (49.6%)	0.786
Quite or very interested in future AI use	34 (82.9%)	47 (94.0%)	31 (81.6%)	112 (86.8%)	0.474

* Agreement was defined as “quite” or “very”.

**Table 3:** Perceived barriers, adoption requirements, and educational needs by professional role *.

Variable	Cath-Lab Director/Department Head	Staff Interventional Cardiologist	Other Roles	Overall	*p* Value
Participants	41	50	38	129	
Perceived barriers					
Medico-legal uncertainty regarding responsibility for errors	22 (53.7%)	19 (38.0%)	17 (44.7%)	58 (45.0%)	0.327
Poor integration with angiography, PACS, and hospital systems	17 (41.5%)	18 (36.0%)	10 (26.3%)	45 (34.9%)	0.361
Cultural resistance or operator distrust	12 (29.3%)	14 (28.0%)	12 (31.6%)	38 (29.5%)	0.935
Insufficient AI-specific training	7 (17.1%)	16 (32.0%)	13 (34.2%)	36 (27.9%)	0.169
Acquisition, licensing, or maintenance costs	8 (19.5%)	18 (36.0%)	8 (21.1%)	34 (26.4%)	0.140
Insufficiently robust clinical evidence	15 (36.6%)	11 (22.0%)	8 (21.1%)	34 (26.4%)	0.197
Doubts regarding validation in real-world populations	6 (14.6%)	10 (20.0%)	15 (39.5%)	31 (24.0%)	0.025
Conditions required for adoption					
Physician able to confirm, modify, or disregard the AI recommendation	24 (58.5%)	27 (54.0%)	25 (65.8%)	76 (58.9%)	0.537
Clear medico-legal framework	18 (43.9%)	22 (44.0%)	17 (44.7%)	57 (44.2%)	0.997
Simple integration with existing clinical systems	22 (53.7%)	19 (38.0%)	11 (28.9%)	52 (40.3%)	0.075
Validation in large, representative populations	14 (34.1%)	21 (42.0%)	14 (36.8%)	49 (38.0%)	0.734
Prospective or randomized evidence of clinical benefit	12 (29.3%)	19 (38.0%)	10 (26.3%)	41 (31.8%)	0.464
Education and training					
No previous training but interested in receiving it	25 (61.0%)	33 (66.0%)	21 (55.3%)	79 (61.2%)	0.392 *
Support for structured inclusion of AI in cardiology training	29 (70.7%)	34 (68.0%)	26 (68.4%)	89 (69.0%)	0.812 *
Practical workshops using clinical cases preferred	12 (29.3%)	21 (42.0%)	17 (44.7%)	50 (38.8%)	0.309
Fellowships or training in expert centers preferred	15 (36.6%)	17 (34.0%)	13 (34.2%)	45 (34.9%)	0.962
Theoretical webinars preferred	7 (17.1%)	13 (26.0%)	2 (5.3%)	22 (17.1%)	0.038

* Multiple selections were permitted for barriers, adoption requirements, and preferred training formats; percentages therefore do not sum to 100%.

**Table 4:** Multivariable logistic regression analysis of factors associated with AI use, trust, and prior education. AI, artificial intelligence; CI, confidence interval; OR, odds ratio.

Outcome Variable/Predictor Variable	Univariable OR (95% CI)	Adjusted OR (95% CI) *	*p* Value
Model 1: current AI use (dependent variable)			
Greater AI familiarity (per category increase)	10.45 (4.12–26.50)	9.91 (3.74–26.26)	<0.001
Age (≥55 vs. <55 years)	0.82 (0.38–1.76)	0.89 (0.34–2.31)	0.812
Female sex	1.45 (0.45–4.68)	1.12 (0.28–4.45)	0.871
Years of experience (>10 vs. ≤10 years)	0.91 (0.42–1.98)	0.78 (0.29–2.10)	0.624
Model 2: good or high overall trust in AI (dependent variable)			
Greater AI familiarity (per category increase)	3.48 (2.02–5.99)	3.26 (1.85–5.74)	<0.001
Professional role (cath-lab director vs. staff/other)	1.38 (0.67–2.85)	1.21 (0.54–2.72)	0.645
Age category (per increasing category)	0.88 (0.64–1.21)	0.94 (0.65–1.36)	0.741
Female sex	1.15 (0.40–3.28)	0.85 (0.26–2.78)	0.789
Model 3: previous participation in AI-focused education (dependent variable)			
Female sex	3.82 (1.21–12.05)	4.90 (1.22–19.78)	0.025
Younger age (per increasing age category)	0.52 (0.28–0.96)	0.47 (0.22–0.99)	0.046
Greater AI familiarity (per category increase)	2.85 (1.58–5.14)	2.68 (1.44–4.99)	0.002
Professional role (staff vs. director/other)	1.24 (0.55–2.78)	1.08 (0.42–2.76)	0.872

* Adjusted for all covariates included in the corresponding multivariable logistic regression model.

## Data Availability

The original contributions presented in this study are included in the article/Supplementary Materials. Further inquiries can be directed to the corresponding author.

## Supplementary Materials

The following supporting information can be downloaded at: https://www.mdpi.com/article/10.3390/jcm15187050/s1, Table S1. Collaborators. Table S2. Original survey. Table S3. Checklist for Reporting Of Survey Studies (CROSS).
