# Supplementary material for: A Survey on Perspectives Toward Artificial Intelligence Among Italian Interventional Cardiologists
Source: J Clin Med. 2026 Sep 11;15(18):7050. doi: 10.3390/jcm15187050 (PMC13607529; doi:10.3390/jcm15187050)
Supplement: Supplementary file 1 [file jcm-15-07050-s001.zip › jcm-4529236-supplementary.pdf]

Online supplement to 'A survey on perspectives toward artificial intelligence among Italian interventional cardiologists', by Giuseppe Biondi-Zoccai et al

Table S1. Collaborators.

Last name	First name	Affiliation
Andò	Giuseppe	Azienda Ospedaliera Papardo, Messina
Angelini	Filippo	Città Della Salute e Della Scienza Hospital, Torino
Arabia	Gianmarco	ASST Spedali Civili Di Brescia, Brescia
Arrivi	Alessio	AOSP "S. Maria", Terni
Asciutto	Salvatore	ARNAS Civico, Palermo
Belmonte	Marta	Azienda Ospedaliero-Universitaria Sant'Andrea, Rome; Sapienza University of Rome, Rome
Bernardi	Marco	Santa Maria Goretti Hospital, Latina
Bocci	Alessandro	Ospedale Maggiore di Lodi, Lodi
Borrello	Bruno	AOU Parma, Parma
Bozzani	Antonio	Fondazione IRCCS Policlinico San Matteo, Pavia
Bruno	Francesco	A.O.U. Città della Salute e della Scienza, Torino
Calvieri	Camilla	Ospedale Cavalieri di Malta, Roma
Casella	Michela	Marche Polytechnic University, Ancona; IRCCS Maria Cecilia Hospital – GVM Care & Research, Cotignola
Casella	Gianni	Ospedale Maggiore, Bologna
Casu	Gavino	Università Sassari, Sassari
Celeski	Mihail	Department of Cardiology, Fabrizio Spaziani Hospital, Frosinone
Cereda	Alberto	Ospedale San Carlo, Milano
Cernetti	Carlo	Ospedale Cà Foncello Treviso
Cinquetti	Martino	Ospedale di Conegliano, Conegliano
Cioppa	Angelo	Clinica Montevergine, Mercogliano
Ciufoli	Michele	Università degli studi di Perugia, Perugia
Cocco	Nino	Policlinico Universitario Campus Biomedico di Roma, Roma
Coluccia	Giovanni	Ospedale "Vito Fazzi", Lecce
Comis	Alessandro	Centro Cuore Morgagni, Pedara
Cortese	Bernardo	Fondazione RIC, Milano

Crimi	Gabriele	IRCCS Maria Cecilia Hospital – GVM Care & Research, Cotignola
D'Alto	Michele	Azienda Ospedaliera dei Colli - Ospedale CTO, Napoli
D'Amico	Gianpiero	Ospedale dell'Angelo, Mestre, Venice
De Candia	Gianfranco	Policlinico Duilio Casula AOU Cagliari, Cagliari
De Michele	Mario	Ospedale Moscati, Aversa
De Rosa	Salvatore	Department of Medical and Surgical Sciences; Magna Graecia University, Catanzaro
Denegri	Andrea	Parma University Hospital, Parma, Italy
Denti	Paolo	Ospedale San Raffaele, Milano
D'Errico	Fabrizio	Ospedale Sant'Eugenio, Roma
Di Gioia	Giuseppe	Clinica Montevergine, Mercogliano
Di Muro	Francesca Maria	Università degli Studi di Salerno, Salerno
Faraci	Alessandro	A.O.U.P. "Paolo Giaccone", Palermo
Fineschi	Massimo	AOUS Siena, Siena
Gallo	Diego	Politecnico di Torino, Torino
Giannico	Maria Benedetta	Ospedale Sant'Eugenio, Rome
Giordan	Massimo	Ospedale di Rovigo, Rovigo
Giusti	Giuliano	ASST Grande Ospedale Niguarda, Cardiologia Pediatrica, Milano
Iacovelli	Fortunato	Policlinico University Hospital Bari, Bari
Lanzone	Saverio	Ospedale Di Venere, Bari
Leoncini	Massimo	Ospedale di Sanremo, Sanremo
Leone	Antonio Maria	Ospedale Isola Tiberina - Gemelli Isola Università Cattolica del Sacro Cuore, Rome
Liga	Riccardo	Azienda Ospedaliero Universitaria Pisana, Pisa
Maffeo	Diego	Fondazione Poliambulanza, Brescia
Magnano	Roberta	ASL Pescara, Pescara
Mancone	Massimo	Policlinico Umberto I, Rome
Mandurino-Mirizzi	Alessandro	Vito Fazzi Hospital, Lecce, Italy, University of Salento, Lecce,
Mangieri	Antonio	IRCCS Humanitas Research Hospital, Rozzano
Marino	Francesco	Ospedale Madre Giuseppina Vannini, Rome
Massaro	Gianluca	Policlinico Tor Vergata, Rome
Mazzone	Patrizio	Ospedale Niguarda, Milano

Menzà	Gregorio	AUSL IRCCS Regio Emilia, Reggio Emilia
Migliore	Giuseppe	PO Villa Sofia, Palermo
Millesimo	Michele	Ospedale di Rivoli, Rivoli
Mingoia	Giulia	ARNAS Civico, Palermo
Mollo	Pasquale	Ospedale Fabrizio Spaziani, Frosinone
Molon	Giulio	IRCCS S. Cuore Don Calabria, Negrar
Montefusco	Antonio	Ospedale Molinette, Torino
Monti	Manuel	USL Umbria 1, Perugia
Moretti	Claudio	Ospedale di Chivasso, Chivasso
Moscucci	Federica	AOU Policlinico Umberto I, Rome, University of L'Aquila, L'Aquila
Muneretto	Claudio	Università di Brescia Spedali Civili, Brescia
Mura	Enrico	HSF ASL 3 Nuoro, Nuoro
Negro	Francesco	Ospedale "Giusppe Fornaroli", Magenta
Palamà	Zefferino	Casa di Cura "Villa Verde", Taranto
Pepe	Martino	Policlinico di Bari, Bari
Petrolini	Alessandro	Aurelia Hospital, Rome
Picchi	Andrea	USL Toscana Sud Est, Grosseto
Pignalberi	Carlo	P.O. San Filippo Neri, Rome
Rubboli	Andrea	Ospedale S. Maria delle Croci, Ravenna
Rubino	Antonino salvatore	Università Kore di Enna, Centro Cuore Morgagni, Pedara
Russo	Filippo maria	Fondazione IRCCS Ca Granda Ospedale Maggiore Policlinico, Milan
Rusticali	Guido	AUSL Piacenza, Piacenza
Scagliola	Riccardo	Ospedale Cardinal G. Massaia, Asti
Sciahbasi	Alessandro	Ospedale Sandro Pertini, Rome
Sciatti	Edoardo	ASST Papa Giovanni XXIII, Bergamo
Sirignano	Pasqualino	Sapienza University of Rome, Rome, Sant'Andrea Hospital, Rome
Soldà	Pier l	Ospedale di Biella, Biella
Sucato	Vincenzo	Azienda Ospedaliera Universitaria Policlinico Paolo Giaccone, Palermo
Tamburino	Claudia	Centro Cuore Morgagni, Catania
Tanzilli	Gaetano	Sapienza Università di Roma, Rome

Tarantini	Giuseppe	University of Padua, Padua
Tavella	Domenico	AOUI Verona, Verona
Tofoni	Paolo	Ospedale Papa Giovanni XXIII, Bergamo
Tomassini	Francesco	Ospedale degli Infermi Rivoli, Rivoli, ASLTO3-AOU San Luigi Gonzaga, Orbassano
Tosi	Paolo	IRCCS Sacro Cuore, Negrar
Trabattoni	Daniela	Centro Cardiologico Monzino, Milan
Trovato	Guglielmo Maria	European Medical Association, Brussels
Tumminello	Gabriele	IRCCS Fondazione Ca' Granda Ospedale Maggiore Policlinico, Milan
Vadala’	Paolo	Ospedale Michele e Pietro Ferrero, Verduno
Verdoia	Monica	Nuovo Ospedale degli Infermi, Biella
Villa	Emmanuel	Fondazione Poliambulanza, Brescia
Vizzari	Giampiero	AOU Policlinico “G. Martino”, Messina
Zezza	Luigi	Ospedale Cardinale G. Panico, Tricase
Zuin	Marco	Padova South Hospitals, Padova

Table S2. Original survey.

Survey sull’intelligenza artificiale in cardiologia interventistica

Descrizione iniziale del modulo

Gentile Collega,

ti invitiamo a partecipare a questa breve **survey sull’utilizzo attuale e sulle prospettive future dell’intelligenza artificiale (IA) nella pratica dei cardiologi interventisti italiani**.

L’obiettivo dell’indagine è valutare il grado di conoscenza, utilizzo, percezione, fiducia e disponibilità all’adozione di strumenti basati sull'IA in cardiologia interventistica. La compilazione richiede circa 5 minuti e le risposte saranno analizzate in forma aggregata e anonimizzata.

La partecipazione è volontaria, ma a tutti i partecipanti saranno comunicati i risultati della survey, e verrà anche offerta l’opportunità di **essere esplicitamente inserito come Contributor negli Acknowledgements** dell’articolo che riassumerà i risultati della survey.

Ti ringraziamo per il Tuo contributo.

Giuseppe Biondi Zoccai, Sapienza Università di Roma e Ospedale Santa Maria Goretti, Latina
Giovanni Vincenzo Biondi Zoccai, Liceo Dante Alighieri, Roma
Fabrizio D'Ascenzo, Università di Torino, Torino

Sezione 1 — Profilo professionale e contesto lavorativo

1. Indirizzo email del partecipante
Tipo domanda: risposta breve
Validazione suggerita: email
Obbligatoria: sì
Campo email: ________

2. Qual è la tua fascia d’età?
Tipo domanda: risposta singola
Obbligatoria: sì

- <35 anni
- 35–44 anni
- 45–54 anni
- 55–64 anni
- ≥65 anni
- Preferisco non rispondere

3. Qual è il tuo ruolo professionale principale?
Tipo domanda: risposta singola
Obbligatoria: sì

- Cardiologo interventista strutturato
- Cardiologo interventista in formazione / fellow
- Responsabile di emodinamica / cath lab director
- Cardiologo clinico con attività in emodinamica
- Altro: ________

4. Da quanti anni svolgi attività in cardiologia interventistica/emodinamica?
Tipo domanda: risposta singola
Obbligatoria: sì

- <5 anni
- 5–10 anni
- 11–20 anni
- 20 anni

5. In quale ospedale/struttura lavori prevalentemente?

Tipo domanda: risposta breve
Obbligatoria: sì
Campo libero: ________
6. In quale città si trova la struttura in cui lavori prevalentemente?
Tipo domanda: risposta breve
Obbligatoria: sì
Campo libero: ________
7. In quale area geografica si trova prevalentemente la struttura in cui lavori?
Tipo domanda: risposta singola
Obbligatoria: sì

- Nord Italia
- Centro Italia
- Sud Italia
- Isole

8. In quale tipologia di struttura lavori prevalentemente?
Tipo domanda: risposta singola
Obbligatoria: sì

- Ospedale pubblico / azienda ospedaliera
- Ospedale universitario / IRCCS
- Ospedale privato accreditato
- Struttura privata non convenzionata
- Altro: ________

9. Come definiresti il profilo principale della tua emodinamica?
Tipo domanda: risposta singola
Obbligatoria: sì

- Centro con emodinamica H24 e rete STEMI
- Centro con emodinamica non H24
- Centro prevalentemente coronarico
- Centro coronarico e strutturale
- Centro ad alto volume con attività complessa coronarica e/o strutturale
- Altro: ________

10. Qual è il volume annuale approssimativo di procedure della tua emodinamica?
Tipo domanda: risposta singola
Obbligatoria: sì

- <500 procedure/anno
- 500–999 procedure/anno
- 1000–1499 procedure/anno
- ≥1500 procedure/anno
- Non so / preferisco non rispondere

Sezione 2 — Familiarità e utilizzo attuale dell'Intelligenza Artificiale
11. Come valuteresti il tuo livello di familiarità con l'Intelligenza Artificiale applicata alla medicina/cardiologia?
Tipo domanda: risposta singola
Obbligatoria: sì

- Nessuna familiarità
- Familiarità bassa
- Familiarità moderata
- Familiarità buona
- Familiarità elevata

12. Attualmente utilizzi strumenti basati su Intelligenza Artificiale nella tua pratica clinica o nella tua attività professionale?
Tipo domanda: risposta singola
Obbligatoria: sì

- Sì, regolarmente
- Sì, occasionalmente
- Sì, ma solo in ambito di ricerca o sperimentazione
- No, ma la mia struttura dispone di strumenti basati su AI
- No, la mia struttura non dispone di strumenti basati su AI
- Non so / non sono sicuro

Logica di salto suggerita per Google Form:
Se la risposta è "Sì, regolarmente", "Sì, occasionalmente" o "Sì, ma solo in ambito di ricerca o sperimentazione", mostrare le domande 13 e 14.
Negli altri casi, passare direttamente alla Sezione 3.

13. In quali ambiti utilizzi attualmente strumenti basati su Intelligenza Artificiale?
Tipo domanda: caselle di controllo
Obbligatoria: sì, solo se mostrata

- Analisi automatica dell'angiografia coronarica
- Valutazione funzionale non invasiva o angiografia-derivata, ad esempio FFR-CT, QFR o analoghi
- Imaging intracoronarico, ad esempio IVUS/OCT con analisi automatizzata
- Pianificazione di procedure coronariche complesse
- Pianificazione di procedure strutturali, ad esempio TAVI, procedure mitraliche, tricuspidi o chiusure percutanee
- Supporto intra-procedurale in tempo reale
- Ottimizzazione della dose radiogena
- Ottimizzazione dell'uso di mezzo di contrasto
- Stratificazione del rischio peri-procedurale
- Predizione di complicanze, ad esempio sanguinamenti, danno renale acuto, restenosi o eventi ischemici
- Refertazione automatizzata o supporto alla documentazione clinica
- Organizzazione del workflow di sala / gestione operativa
- Ricerca scientifica e analisi dati
- Formazione e simulazione
- Altro: ________

14. Nel complesso, come valuti l'impatto degli strumenti basati su Intelligenza Artificiale che hai utilizzato finora?
Tipo domanda: griglia a scelta multipla
Obbligatoria: sì, solo se mostrata
Scala:
1 = impatto molto negativo / nullo
2 = impatto basso
3 = impatto moderato
4 = impatto positivo
5 = impatto molto positivo
Valutare i seguenti aspetti:

- Qualità diagnostica
- Pianificazione procedurale
- Sicurezza della procedura
- Efficienza del workflow
- Riduzione dei tempi

- Supporto alla decisione clinica
- Fiducia dell’operatore nello strumento
- Facilità di integrazione nella pratica quotidiana

Sezione 3 — Aree di potenziale applicazione futura
15. In quali ambiti ritieni che l’Intelligenza Artificiale possa avere il maggiore impatto futuro in emodinamica?
Tipo domanda: caselle di controllo
Suggerimento: massimo 3 risposte
Obbligatoria: sì

- Diagnosi e quantificazione automatica delle stenosi coronariche
- Valutazione funzionale delle lesioni coronariche
- Integrazione tra angiografia, imaging intracoronarico e dati clinici
- Pianificazione di PCI complesse
- Pianificazione di procedure strutturali
- Guida intra-procedurale in tempo reale
- Predizione del rischio individuale del paziente
- Prevenzione e gestione delle complicanze
- Ottimizzazione di radiazioni e mezzo di contrasto
- Automazione della refertazione
- Ottimizzazione del workflow di sala
- Formazione, simulazione e training degli operatori
- Ricerca clinica e generazione di registri
- Altro: ________

16. Secondo te, nei prossimi 3–5 anni l’Intelligenza Artificiale in emodinamica sarà:
Tipo domanda: risposta singola
Obbligatoria: sì

- Uno strumento standard in molte procedure
- Uno strumento utile soprattutto nei casi complessi
- Uno strumento limitato a centri ad alto volume o accademici
- Uno strumento prevalentemente di ricerca
- Uno strumento marginale nella pratica clinica
- Non so

17. Quanto sei d’accordo con le seguenti affermazioni?
Tipo domanda: griglia a scelta multipla
Obbligatoria: sì
Scala:
1 = per nulla d’accordo
2 = poco d’accordo
3 = neutrale
4 = abbastanza d’accordo
5 = molto d’accordo
Valutare le seguenti affermazioni:

- L’Intelligenza Artificiale può migliorare la standardizzazione delle procedure in emodinamica.
- L’Intelligenza Artificiale può aumentare la precisione diagnostica e procedurale.
- L’Intelligenza Artificiale può contribuire a ridurre tempi procedurali, radiazioni o mezzo di contrasto.
- L’Intelligenza Artificiale può migliorare la selezione dei pazienti candidati a procedure interventistiche.
- L’Intelligenza Artificiale può supportare la formazione dei giovani cardiologi interventisti.

- L’Intelligenza Artificiale dovrebbe restare uno strumento di supporto e non sostituire il giudizio del medico.
- L’Intelligenza Artificiale potrebbe modificare significativamente il ruolo del cardiologo interventista nei prossimi anni.
- Mi sentirei a mio agio nell’utilizzare raccomandazioni generate da un sistema AI, purché supervisionate dal medico.

Sezione 4 — Barriere, criticità e fiducia
18. Quali sono, secondo te, le principali barriere alla diffusione dell’Intelligenza Artificiale nelle emodinamiche italiane?
Tipo domanda: caselle di controllo
Suggerimento: massimo 3 risposte
Obbligatoria: sì

- Costi elevati di acquisizione, licenza o manutenzione
- Scarsa integrazione con angiografi, PACS, cartella clinica o sistemi ospedalieri
- Mancanza di formazione specifica per medici e personale di sala
- Mancanza di evidenze cliniche sufficientemente solide
- Dubbi sulla validazione degli algoritmi nella popolazione reale
- Problemi di privacy, gestione dei dati o cybersecurity
- Incertezza medico-legale sulla responsabilità in caso di errore
- Resistenza culturale o diffidenza degli operatori
- Mancanza di supporto amministrativo o direzionale
- Tempi burocratici per acquisto e implementazione
- Timore di riduzione dell’autonomia decisionale del medico
- Altro: ________

19. Qual è il tuo livello di fiducia complessivo negli strumenti basati su Intelligenza Artificiale applicati all’emodinamica?
Tipo domanda: risposta singola
Obbligatoria: sì

- Nessuna fiducia
- Bassa fiducia
- Fiducia moderata
- Buona fiducia
- Elevata fiducia
- Non so

20. Quali condizioni ritieni necessarie prima di adottare stabilmente uno strumento basato su Intelligenza Artificiale nella pratica clinica?
Tipo domanda: caselle di controllo
Obbligatoria: sì

- Validazione clinica su popolazioni ampie e rappresentative
- Studi prospettici o randomizzati che dimostrino beneficio clinico
- Approvazione regolatoria chiara
- Integrazione semplice con i sistemi già in uso
- Trasparenza sul funzionamento dell’algoritmo
- Possibilità per il medico di confermare, modificare o ignorare il suggerimento dell’AI
- Formazione pratica degli operatori
- Supporto tecnico continuo
- Chiarezza sugli aspetti medico-legali
- Valutazione costo-efficacia
- Altro: ________

Sezione 5 — Formazione e disponibilità all'adozione
21. Hai mai partecipato a corsi, webinar o eventi formativi sull'Intelligenza Artificiale applicata alla cardiologia o all'emodinamica?
Tipo domanda: risposta singola
Obbligatoria: sì

- Sì, più volte
- Sì, una volta
- No, ma sarei interessato
- No, e al momento non sono interessato

22. Ritieni che la formazione sull'Intelligenza Artificiale debba essere inclusa nei percorsi formativi dei cardiologi interventisti?
Tipo domanda: risposta singola
Obbligatoria: sì

- Sì, come parte strutturata del training
- Sì, ma solo come formazione opzionale
- Non so
- No, non la ritengo prioritaria
- No

23. Quale formato formativo sarebbe più utile per te?
Tipo domanda: caselle di controllo
Suggerimento: massimo 2 risposte
Obbligatoria: sì

- Webinar teorici
- Workshop pratici su casi clinici
- Sessioni hands-on durante congressi
- Corsi ECM dedicati
- Moduli online brevi
- Fellowship o training specifici in centri esperti
- Materiale pratico su software e casi reali
- Altro: ________

24. Quanto saresti interessato a utilizzare strumenti basati su Intelligenza Artificiale nella tua pratica nei prossimi anni?
Tipo domanda: risposta singola
Obbligatoria: sì

- Molto interessato
- Abbastanza interessato
- Neutrale
- Poco interessato
- Per nulla interessato

Sezione 6 — Domande aperte finali
25. Quale applicazione dell'Intelligenza Artificiale ritieni più promettente per il futuro dell'emodinamica?
Tipo domanda: risposta aperta breve
Obbligatoria: no
Campo libero: ________
26. Qual è la tua principale preoccupazione riguardo all'introduzione dell'Intelligenza Artificiale in emodinamica?
Tipo domanda: risposta aperta breve
Obbligatoria: no
Campo libero: ________

27. Hai ulteriori commenti, suggerimenti o esperienze da condividere sull'utilizzo dell'Intelligenza Artificiale in cardiologia interventistica?
Tipo domanda: paragrafo
Obbligatoria: no
Campo libero: ________

Sezione 7 — Risultati della survey e Acknowledgements
28. Desideri ricevere i risultati aggregati della survey quando saranno disponibili?
Tipo domanda: risposta singola
Obbligatoria: sì

- Sì
- No

Nota: in caso di risposta "Sì", i risultati saranno inviati all'indirizzo email indicato all'inizio del modulo.
29. Desideri essere citato negli Acknowledgements dell'eventuale articolo scientifico derivato da questa survey?
Tipo domanda: risposta singola
Obbligatoria: sì

- Sì, desidero essere citato negli Acknowledgements
- No, preferisco non essere citato

Logica di salto suggerita per Google Form:
Se la risposta è "Sì, desidero essere citato negli Acknowledgements", mostrare le domande 30–32.
Se la risposta è "No, preferisco non essere citato", passare direttamente al messaggio conclusivo.
30. Nome e cognome da riportare negli Acknowledgements
Tipo domanda: risposta breve
Da mostrare solo se alla domanda 29 viene selezionato "Sì"
Obbligatoria: sì, solo se mostrata
Campo libero: ________
31. Affiliazione/ospedale da riportare negli Acknowledgements
Tipo domanda: risposta breve
Da mostrare solo se alla domanda 29 viene selezionato "Sì"
Obbligatoria: sì, solo se mostrata
Esempio: U.O. Cardiologia Interventistica, Nome Ospedale, Città
Campo libero: ________
32. Confermi di autorizzare l'uso del tuo nome, cognome e affiliazione negli Acknowledgements dell'eventuale articolo scientifico derivato dalla survey?
Tipo domanda: risposta singola
Da mostrare solo se alla domanda 29 viene selezionato "Sì"
Obbligatoria: sì, solo se mostrata

- Sì, confermo
- No, preferisco non essere citato

Messaggio conclusivo
Grazie per aver completato la survey.
Il tuo contributo sarà utile per comprendere lo stato attuale e le prospettive future dell'Intelligenza Artificiale nelle emodinamiche italiane e nella pratica dei cardiologi interventisti italiani.
Le risposte saranno analizzate in forma aggregata. L'indirizzo email sarà utilizzato per eventuali comunicazioni relative alla survey, inclusa la trasmissione dei risultati aggregati qualora richiesta dal partecipante. L'eventuale citazione negli Acknowledgements avverrà solo in caso di esplicita autorizzazione.

Table S3. Checklist for Reporting Of Survey Studies (CROSS).

Section/topic	Item	Item description	Reported on page #
**Title and abstract**			
Title and abstract	1a	State the word "survey" along with a commonly used term in title or abstract to introduce the study's design.	1
	1b	Provide an informative summary in the abstract, covering background, objectives, methods, findings/results, interpretation/discussion, and conclusions.	3
**Introduction**			
Background	2	Provide a background about the rationale of study, what has been previously done, and why this survey is needed.	5-6
Purpose/aim	3	Identify specific purposes, aims, goals, or objectives of the study.	6
**Methods**			
Study design	4	Specify the study design in the methods section with a commonly used term (e.g., cross-sectional or longitudinal).	7
	5a	Describe the questionnaire (e.g., number of sections, number of questions, number and names of instruments used).	7-8
Data collection methods	5b	Describe all questionnaire instruments that were used in the survey to measure particular concepts. Report target population, reported validity and reliability information, scoring/classification procedure, and reference links (if any).	7-8
	5c	Provide information on pretesting of the questionnaire, if performed (in the article or in an online supplement). Report the method of pretesting, number of times questionnaire was pre-tested, number and demographics of participants used for pretesting, and the level of similarity of demographics between pre-testing participants and sample population.	7-8
	5d	Questionnaire if possible, should be fully provided (in the article, or as appendices or as an online supplement).	7-8
Sample characteristics	6a	Describe the study population (i.e., background, locations, eligibility criteria for participant inclusion in survey, exclusion criteria).	7
	6b	Describe the sampling techniques used (e.g., single stage or multistage sampling, simple random sampling, stratified sampling, cluster sampling, convenience sampling). Specify the locations of sample participants whenever clustered sampling was applied.	7
	6c	Provide information on sample size, along with details of sample size calculation.	9
	6d	Describe how representative the sample is of the study population (or target population if possible), particularly for population-based surveys.	14
Survey administration	7a	Provide information on modes of questionnaire administration, including the type and number of contacts, the location where the survey was conducted (e.g., outpatient room or by use of online tools, such as SurveyMonkey).	7-8
	7b	Provide information of survey's time frame, such as periods of recruitment, exposure, and follow-up days.	7

	7c	Provide information on the entry process:<br>–>For non-web-based surveys, provide approaches to minimize human error in data entry.<br>–>For web-based surveys, provide approaches to prevent "multiple participation" of participants.	7
Study preparation	8	Describe any preparation process before conducting the survey (e.g., interviewers' training process, advertising the survey).	7
Ethical considerations	9a	Provide information on ethical approval for the survey if obtained, including informed consent, institutional review board [IRB] approval, Helsinki declaration, and good clinical practice [GCP] declaration (as appropriate).	7-8
	9b	Provide information about survey anonymity and confidentiality and describe what mechanisms were used to protect unauthorized access.	7-8
Statistical analysis	10a	Describe statistical methods and analytical approach. Report the statistical software that was used for data analysis.	8
	10b	Report any modification of variables used in the analysis, along with reference (if available).	21, 23-24
	10c	Report details about how missing data was handled. Include rate of missing items, missing data mechanism (i.e., missing completely at random [MCAR], missing at random [MAR] or missing not at random [MNAR]) and methods used to deal with missing data (e.g., multiple imputation).	6
	10d	State how non-response error was addressed.	14
	10e	For longitudinal surveys, state how loss to follow-up was addressed.	6
	10f	Indicate whether any methods such as weighting of items or propensity scores have been used to adjust for non-representativeness of the sample.	6
	10g	Describe any sensitivity analysis conducted.	6
**Results**			
Respondent characteristics	11a	Report numbers of individuals at each stage of the study. Consider using a flow diagram, if possible.	7, 9
	11b	Provide reasons for non-participation at each stage, if possible.	7
	11c	Report response rate, present the definition of response rate or the formula used to calculate response rate.	7
	11d	Provide information to define how unique visitors are determined. Report number of unique visitors along with relevant proportions (e.g., view proportion, participation proportion, completion proportion).	7
Descriptive results	12	Provide characteristics of study participants, as well as information on potential confounders and assessed outcomes.	9-11, 19-24
Main findings	13a	Give unadjusted estimates and, if applicable, confounder-adjusted estimates along with 95% confidence intervals and p-values.	23-24

	13b	For multivariable analysis, provide information on the model building process, model fit statistics, and model assumptions (as appropriate).	8
	13c	Provide details about any sensitivity analysis performed. If there are considerable amount of missing data, report sensitivity analyses comparing the results of complete cases with that of the imputed dataset (if possible).	8
**Discussion**			
Limitations	14	Discuss the limitations of the study, considering sources of potential biases and imprecisions, such as non-representativeness of sample, study design, important uncontrolled confounders.	14
Interpretations	15	Give a cautious overall interpretation of results, based on potential biases and imprecisions and suggest areas for future research.	12-14
Generalizability	16	Discuss the external validity of the results.	14
**Other sections**			
Role of funding source	17	State whether any funding organization has had any roles in the survey's design, implementation, and analysis.	2
Conflict of interest	18	Declare any potential conflict of interest.	2
Acknowledgements	19	Provide names of organizations/persons that are acknowledged along with their contribution to the research.	2, 8; S1-S4
